# The Immunostimulatory Effects of Commercial Feed Additives on Growth Performance, Non-specific Immune Response, Antioxidants Assay, and Intestinal Morphometry of Nile tilapia, *Oreochromis niloticus*

**DOI:** 10.3389/fphys.2021.627499

**Published:** 2021-02-25

**Authors:** Mohamed Ibrahim Kord, Tarek Mohamed Srour, Eglal Ali Omar, Ahmed Awny Farag, Abdel Aziz Moussa Nour, Hala Saber Khalil

**Affiliations:** ^1^Central Laboratory for Agricultural Climate, ARC, Giza, Egypt; ^2^Department of Animal and Fish Production, Faculty of Agriculture (Saba Basha), Alexandria University, Alexandria, Egypt; ^3^WorldFish, Africa Aquaculture Research and Training Center, Abbassa, Egypt; ^4^National Institute of Oceanography and Fisheries (NIOF), Cairo, Egypt

**Keywords:** immunostimulants, phagocytic index, lysozyme, antioxidant enzyme, intestinal morphology

## Abstract

The main objective of the present research was to investigate the impacts of commercial immunostimulants on growth, non-specific immune response, antioxidant enzymes, and intestinal morphometry of Nile tilapia, *Oreochromis niloticus*. Fish (100 ± 6.5 g) were randomly divided into five groups in triplicates (150 fish in each replicate), stocked in 20 m^2^ of aerated concrete ponds. The fish were fed on a control diet (30.0% crude protein) (control), and four experimental diets supplemented with Yeast Plus^®^, Digestarom^®^, and Biotronic^®^ Top^3^ at 1 kg ton^–1^, and Sanolife PRO-F^®^ at 0.5 kg ton^–1^. After the experimental period, the highest significant yield (kg m^–3^), mean final weight (g fish^–1^), average weight gain (g fish^–1^), and specific growth rate (% body weight day^–1^), were recorded in fish fed on the diet supplemented by Sanolife PRO-F, followed by Yeast Plus ponds. The lowest yield was recorded in the control group. However, the feed conversion ratio was significantly decreased with Sanolife PRO-F diet. Furthermore, the hematological analysis increased in the following ascending order: Sanolife PRO-F^®^; Yeast Plus^®^; Biotronic^®^ Top3 and finally Digestarom^®^ groups. The lowest concentrations of white blood cells, red blood cells, hematocrite, and hemoglobin (*P* ≤ 0.05) were observed in the control group. The levels of phagocytic activity and phagocytic index were significantly higher in fish fed with Sanolife PRO-F^®^ group (*P* ≤ 0.05). Likewise, serum lysozyme activity was significantly highest in Sanolife PRO-F (0.63 and 0.68 U/mL, after 0.5 min and 3 min, respectively). Levels of total serum proteins, globulin, Immunoglobulin M, catalase, and super oxide dismutase were significantly higher in fish fed with Sanolife PRO-F^®^ supplement. On the contrary, length measurement of the intestinal villus height/width, absorption area, crypt depth, and goblet cells, were significantly lower in the control group, whereas their highest values was observed in fish fed Sanolife PRO-F (*P* ≤ 0.05). Consequently, Sanolife PRO-F^®^ is recommended at a level of 0.5 kg ton^–1^, to improve the growth performance, antioxidative capacity, and immune response of Nile tilapia.

## Graphical Abstract

The immunostimulatory effects of commercial feed additives on growth performance, non-specific immune response, antioxidants assay, and intestinal morphometry of Nile tilapia, *O. niloticus*.

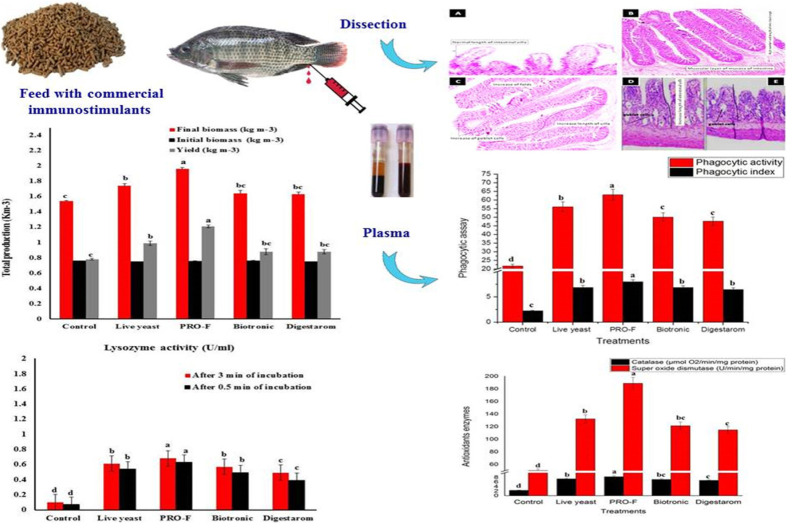

## Introduction

In fact, the sustainability of fish farming has been changed over the past years through intensive cultivation methods. Sustainable aquaculture development strategies are supported by assessing the effectiveness of commercial immunostimulants (prebiotics and probiotics as well as organic acids), to explore their effects for promoting growth, survival present, improving the non-specific immune response, antioxidants assay, and maintaining of intestine health (Newaj-Fyzul and Austin, 2015; Khalila et al., 2018; Ismail and Al-Hamdani, 2019).

Immunostimulant plays an essential role in increasing the growth performance of aquatic organisms via improving the efficiency of feed digestion and utilization (Ringø et al., 2012). Probiotics supplements are identified as living microorganisms from gram-negative or positive bacteria, fungi, and algae that can improve the nutritional content of the feed used to feed fish when added in appropriate quantities (Oliveira et al., 2017). Scientists used commercial probiotics, such as; *Bacillus spp., Streptococcus*, *Lactococcus lactis, Pseudomonas fluorescens, Vibrio alginolyticus, Saccharomyces cerevisiae*, *Debrayomyces hansenii, Spirulina*, to enhance growth performance and immune response over past years in juvenile Nile tilapia, *Oreochromis niloticus* (Abdel-Tawwab et al., 2008; Abdel-Tawwab and Ahmad, 2009; Abdel-Tawwab, 2012). They give many benefits, such as increasing the rate of nutritional conversion of feed, improving growth efficiency via improving the balance of gastrointestinal microbial communities, as well as increasing the creation of supplemental digestive enzymes and increasing immune system responses, against pathogenic bacteria by producing inhibitory compounds (Bozkurt et al., 2014; Amenyogbe et al., 2020). Besides, the beneficial effect of organic acids as a functional feed additive on digestion track, gut microbiota and morphology, gut pH, growth, nutrient utilization, feed palatability, and hepatopancreas pathophysiology for Nile tilapia, *O. niloticus* (Ng and Koh, 2017; Abd El-Naby et al., 2019; Abdel-Tawwab et al., 2020a). Elsabagh et al. (2018) and Amenyogbe et al. (2020) stated that the addition of commercial immunostimulants in aquatic-feeds is expected to increase substantially in the coming years due to changing global regulatory controls that attempt to provide more sustainable aquaculture via enhancing growth capacity, feeding efficiency, and immune responses as well as reducing the intestinal inflammation in Nile tilapia, *O. niloticus.*

Nile tilapia, *O. niloticus*, became one of the most important freshwater fish in Egypt and many other countries all over the world (El-Sayed, 2019; Allam et al., 2020). The present study attempts to link between the feed additives industry sector and the sustainability of applied aquaculture strategies and farm biosecurity by evaluating commercial immunostimulants (prebiotics and probiotics as well as organic acids) to find out which one is the most effective to apply and thus to improve the growth performance. Therefore, the current study was performed to explore the impacts of commercial immunostimulants (Yeast Plus^®^, Sanolife PRO-F^®^, Biotronic^®^ Top^3^, and Digestarom^®^) on total production per cubic meter, growth efficiency, non-specific immune response, antioxidants assay, and maintenance of intestine health of Nile tilapia, *O. niloticus.*

## Materials and Methods

### Experimental Diets

The control diet consists of 30% crude protein and 4% crude lipid to meet the requirements of Nile tilapia. One of the four feed additives were added to 1 kg of the control diet, prepared according to the guides of the manufacturer, at levels of 0.5 g feed Sanolife PRO-F^®^, 1 g Yeast Plus^®^, 1 g Biotronic^®^ Top^3^, and 1 g Digestarom^®^. All diets were prepared by mixing the feed ingredients with 100 ml of water thoroughly. After that, ingredients are placed in a pelletizer on die 4 mm diameter, and then dried in the oven at 60°C. Diets were kept in the refrigerator (4°C) until use. Ingredients and proximate chemical composition of the experimental diets presented in Table 1.

**TABLE 1:** Ingredients and proximate chemical composition of the experimental diet without supplementation (g kg^–1^ on dry matter basis).

Feed ingredients	Composition and chemical analysis
Fish meal (65%)	160
Yellow corn	280
Soybean meal (40%)	400
Wheat bran	100.5
Soybean oil	20.5
Distiller’s dried grain with solubles	9
Vitamins and Minerals mixture^1^	30
Total	1000
Chemical analysis (on dry matter basis)	
Dry matter (Dm) %	922.8
Crude protein (CP) %	301.8
Ether extract (EE) %	44.4
Crude fiber (CF) %	93.3
Ash %	101.2
NFE^2^ %	459.3
Me (Kcal/Kg diet)^3^	2610
P/E ratio^4^	115.6

*^1^Vitamin and mineral mixture/kg premix: vitamin D3, 0.8 million IU; A, 4.8 million IU; E, 4 g; K, 0.8 g; BI, 0.4 g; Riboflavin, 1.6 g; B6, 0.6 g; B12, 4 mg; pantothenic acid, 4 g; Nicotinic acid, 8 g; Folic acid, 0.4 g Biotin, 20 mg, Mn, 22 g; Zn, 22 g; Fe, 12 g; Cu, 4 g; I, 0.4 g; Selenium, 0.4 g and Co, 4.8 mg.*

*^2^Nitrogen free extract (NFE) = 100 – (CP + EE + CF+ Ash).*

*^3^Metabolizable energy was calculated from ingredients based on NRC (2011) values for tilapia.*

*^4^Protein to energy ration in mg protein/Kcal ME.*

### Feed Supplements

Yeast Plus^®^ produced by Angel Yeast Egypt Company Ltd., Egypt, which contains *Saccharomyces cerevisiae* (2.21 × 10^12^ CFU/g), *Lactobacillus sp.* (2 × 10^12^ CFU/g); the recommended dose is 1 kg ton^–1^. Sanolife PRO-F^®^ is a blend of *Bacillus* strains, i.e., *Bacillus subtilis* (3.25 × 10^9^ CFU/g), *Bacillus licheniformis* (3.50 × 10^9^ CFU/g), *Bacillus pumilus* (3.25 × 10^9^ CFU/g) produced by INVE Aquaculture, Belgium, with a total number 1.0 × 10^10^ CFU/g, the recommended dose is 0.5 kg ton^–1^. Biotronic^®^ Top^3^ produced by Biomin GmbH, Austria, which contains 99 g formic acid (85%), 145 g ammonium formate (35%), 100 g acetic acid (99.5%), 50 g propionic acid (99.5%), 40 g fumaric acid (85%), and vermiculite up to 1 kg; the recommended dose is 1 kg ton^–1^. Digestarom^®^ as digestive enzymes produced by Biomin, GmbH, Austria, that contained active components: 1-Carvon, Anethole, and 1-Menthol, used in the diet of aquatic animals, like shrimp and fish; the recommended dose is 1 kg ton^–1^.

### Experimental Fish and Facilities

Healthy Nile tilapia fingerlings were obtained from the nursery earthen pond in a private fish farm in Kafr-El-Sheikh governorate. Fish were stocked in 20 m^–3^ concrete ponds (2.5 m width × 8 m length × 0.90 m height), fed on the control diet (30% crude protein), and acclimatized for 14 days prior to the experiment. After acclimation, fish (100 ± 6.5 g) were randomly distributed into fifteen aerated concrete ponds at a density of 50 fish pond^–1^. The experimental ponds were divided into five treatments with three triplicate groups for each. Ponds were siphoned and supplied with a running freshwater before the first feeding.

Fish in each pond were fed on 3% of the total live body weight of the fish, and were regulated properly according to Eurell et al. (1978). Every 2 weeks, the total fish consumption in each pond were collected, weighted and the total amount of feed consumed by the fish was adjusted accordingly. The daily amount of feed was administrated 6 days week^–1^, two equal meals per day, for 8 weeks on two occasions (9.00 am and 3.00 pm). Moreover, fish mortality was recorded, and thus the quantity of feed was readjusted accordingly.

### Physico-Chemical Analyses of Water Samples

Water samples were taken from each experimental pond for quality evaluation during 8 weeks. The pH, temperature (°C), and dissolved oxygen (mg L^–1^) were measured using a multiparametric equipment HANNA (HI 9829). In the meantime, nitrogen compounds, such as unionized ammonia (NH_3_; mg L^–1^), nitrites (NO_2_; mg L^–1^), and nitrates (NO_3_; mg L^–1^) were analyzed and measured using a DREL spectrophotometer 2000 (HACH Company, Loveland, CO, United States) (Boyd, 1990).

### Calculated Parameters

Growth performance was determined at the end of the experiment (8 weeks), including total biomass gain (TBG), weight gain (IAWG, g), feed conversion ratio (FCR), and fish survival (%) were calculated as follows:

Total biomass gain (yield; kg m^–3^) = [(Final biomass _58_ – Initial biomass _0_) / water volume (m^3^)];
Individual weight gain (IWG, g fish^–1^) = [(FW – IW_0_) / number of fish];
Specific growth rate (SGR; %/day) = 100 [Ln (FW) – Ln (IW_0_)] / days of the experiment;
Feed conversion ratio (FCR) = [Feed intake (g) / Weight gain (g)];
Fish survival (%) = 100 [(Initial Population – Final Population) / Initial Population].

### Bleeding and Serum Samples Collection

At the end of the experiment, prior to weighing and blooding samples were taken, the fish were anesthetized using clove oil (5 mL L^–1^), five fish from each pond were anesthetized and blood samples were drawn from through the caudal vessels of the treated fish. Another portion of blood sample was drawn with a syringe containing anticoagulants (0.1 ml of sodium citrate solution) and used to determine white blood cells (WBC), red blood cells (RBC), hematocrit (Ht), and hemoglobin (Hb) as well as phagocytosis. The remaining blood sample was transferred to Eppendorf tubes, without anticoagulant, and centrifuged for 10 min at 2000 × *g*. Serum was collected by a micropipette, and then stored in Eppendorf tubes at −20°C until blood parameters, serum lysozyme activity, and different immune parameters as well as different biomarkers were analyzed.

### Determination of Hematological Parameters

Total red blood cell (RBCs) and white blood cell (WBCs) count as well as the packed cell volume (PCV), were determined manually using a Neubauer’s haemocytometer with Hayem solution as diluent. WBC percentages were determined by counting 1500 and 200 cells, respectively. Hemoglobin concentration (Hb) was determined via using a hemoglobin reagent set (Zeist Chem Diagnostics), according to the cyanomethaemoglobin method of Drabkin (1945).

### Determination of Phagocytic Activity and Index

Yeast strain: The department of poultry and fish diseases, Faculty of Veterinary Medicine, Alexandria University, was supplied *Candida albicans* strain that was used for phagocytic assay study according to Kawahara et al. (1991).

Phagocytic activity (PA) = phagocytic cells % containing yeast cells.
Phagocytic index (PI) = Yeast cells phagocytized numbers / Phagocytic cells numbers.

### Determination of Immunological Parameters

Total proteins in fish serum as well as serum albumin were determined according to Doumas et al. (1981), using commercial kits produced by Pasteur, Lab of Chemroy. Serum globulin was determined by subtracting the total serum albumin from total serum protein according to Kumar et al. (2005). Total Immunoglobulin M assay was conducted in fish serum according to the previously mentioned procedure Milla et al. (2010).

Serum lysozyme activity was determined according to Ellis (1990), methodology using a *Micrococcus lysodeikticus* lysis. In brief, 0.02% of *M. lysodeikticus* was lyophilized at 0.05 mM, using phosphate buffer (pH 6.4) as a substrate, 10 μL of fish serum was added to 250 μL of bacterial suspension in duplicate wells of a U-bottom microtitre plate and absorbance reduction was determined at 480 nm, after 0.5 and 4.5 min of incubation at 23°C, using a microplate reader.

### Determination of Antioxidants Enzymes in Serum

Catalase activity was determined according to Claiborne (1985) methodology, using a mixture of 19 mM hydrogen peroxide, 50 mM of potassium phosphate buffer, 10% PMS and 7.4 pH, under 25°C, while the absorption change was recorded at 240 nm. Super oxide dismutase activity was determined according to Payá et al. (1992) methodology, with some modifications Peixoto et al. (2006), using a detection molecule instead of cytochrome *c*, as nitrotetrazolium blue chloride (NBC), via mixture of (10 mM) hypoxanthine, (10 mM) NBT, and (100 mM, pH 7.0) potassium phosphate. The reaction was performed by control to inhibit 50% reduction of nitrotetrazolium blue chloride.

### Intestinal Morphometric Analysis

The intestinal tract was collected from the fish of the experimental groups as well as the control group and rapidly placed in a 10% formalin buffer for 24 h. From each part, fifteen sections were subjected to histomorphometric analysis, in order to estimate the intestinal perimeter ratio (arbitrary units; AU) according to Dimitroglou et al. (2009). The villi length/width were determined, and the number of intestinal goblet cells was counted and the average number of goblet cells was determined according to Samanya and Yamauchi (2002).

### Statistical Analysis

The data obtained were analyzed statistically via a fully randomized design by one-way ANOVA, using SPSS (Standard version 17.0; SPSS, Chicago, IL, United States). Tukey’s test was used as a *post-hoc* test to analyze the variations between mean values at *p* ≤ 0.05 levels (Dytham, 2011). Values were presented as the mean value ± SE of replicates.

## Results

### Physico-Chemical Parameters of Water Quality

The mean values of water quality parameters were 5.47 ± 0.31 mg L^–1^ for DO, 7.62 ± 0.27 for pH, 0.03 ± 0.001 mg L^–1^ for unionized ammonia, 0.31 ± 0.029 mg L^–1^ for nitrite, and 3.17 ± 0.37 mg L^–1^ for nitrate on day zero. Water quality parameters at the end of the experiment are shown in Table 2. The ponds were supplemented by Sanolife PRO-F^®^ and Yeast Plus^®^ in the feeds, and showed improvements in all water quality parameters compared to Biotronic^®^, Digestarom^®^ and control ponds.

**TABLE 2:** Effect of commercial immunostimulants on water quality parameters in different groups of Nile tilapia, *O. niloticus* after 8 weeks.

Items	Control	Yeast Plus^®^	Sanolife PRO-F^®^	Biotronic^®^ Top^3^	*Digestarom*^®^
D. O.	5.17^*d*^ ± 0.13	5.34^*b*^ ± 0.22	5.39^*a*^ ± 0.13	5.27^*c*^ ± 0.31	5.29^*c*^ ± 0.13
pH	7.72^*a*^ ± 0.15	7.66^*a*^ ± 0.31	7.78^*a*^ ± 0.39	7.44^*a*^ ± 0.17	7.38^*a*^ ± 0.22
NH_3_	0.08^*a*^ ± 0.002	0.04^*d*^ ± 0.002	0.01^*e*^ ± 0.002	0.05^*b*^ ± 0.003	0.03^*c*^ ± 0.001
NO_2_^–^	0.38^*a*^ ± 0.017	0.28^*c*^ ± 0.021	0.25^*d*^ ± 0.027	0.30^*b*^ ± 0.018	0.27^*c*^ ± 0.026
NO_3_^–^	4.19^*a*^ ± 0.27	3.78^*d*^ ± 0.28	3.65^*e*^ ± 0.36	3.87^*b*^ ± 0.27	3.77^*c*^ ± 0.33

*D.O, Dissolved oxygen (mg/l); pH; NH_3_, Unionized ammonia mg L^–1^; NO_2_^–^, Nitrite (mg L^–1^); NO_3_^–^, Nitrate (mg L^–1^). Data represented as means ± SE (*n* = 3). Within rows, means with different superscripts a, b, c, and d indicating that their corresponding means are significantly different at *P* < 0.05.*

### Growth Performance and Feed Utilization Parameters

**FIGURE 1:**
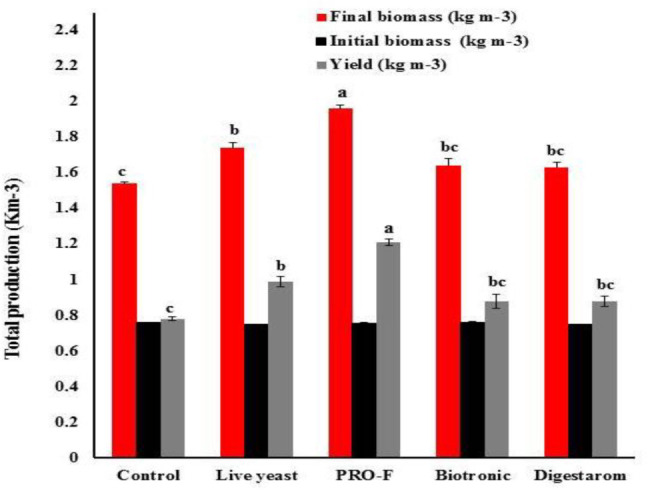
Total production of Nile tilapia, *O. niloticus* fed diets with commercial immunostimulants for 8 weeks. Bars superscripted by different letters within the same axis are significantly different (*P* < 0.05), (*n* = 3; means ± SE).

After 8 weeks of feeding trial, there were significant changes in fish growth indices and total production of Nile tilapia (*P* < 0.05; Figure 1 and Table 3). The highest significant yield (kg m^–3^), final average weight (g fish^–1^), IWG (g fish^–1^), and SGR (% body weight day^–1^) were recorded in fish fed on the diet supplemented by Sanolife PRO-F^®^ followed by that received Yeast Plus^®^, while the lowest performance was observed on the control group. However, the feed conversion ratio was significantly decreased with the Sanolife PRO-F diet. There were no significant differences in the fish survival among all treatments.

**TABLE 3:** Effect of commercial immunostimulants on growth performance of Nile tilapia, *O. niloticus* for 8 weeks.

Items	Control	Yeast Plus^®^	Sanolife PRO-F^®^	Biotronic^®^ Top^3^	Digestarom^®^
Initial average weight (g)	101.33 ± 2.02	100.00 ± 2.88	101.00 ± 2.08	101.66 ± 4.40	100.00 ± 2.88
Final average weight (g)	224.66^*d*^ ± 1.20	246.00^*b*^ ± 2.64	271.66^*a*^ ± 1.76	236.66^*c*^ ± 2.33	234.66^*c*^ ± 2.60
Individual weight gain	123.33^*d*^ ± 2.18	146.00^*b*^ ± 0.57	170.66^*a*^ ± 0.33	135.00^ c^ ± 4.58	134.66^*c*^ ± 2.60
Specific growth rate (%/day)	1.42^*c*^ ± 0.03	1.60^*b*^ ± 0.03	1.76^*a*^ ± 0.02	1.51^*bc*^ ± 0.07	1.52^*bc*^ ± 0.04
Total feed intake (g feed pond^–1^)	223.33^*b*^ ± 3.33	237.66^*ab*^ ± 7.66	248.66^*a*^ ± 1.33	236.66^*ab*^ ± 3.75	245.66^*a*^ ± 2.96
Feed conversion ratio	1.42^*a*^ ± 0.05	1.20^*b*^ ± 0.02	1.02^*c*^ ± 0.03	1.34^*a*^ ± 0.05	1.38^*a*^ ± 0.05
Fish survival (%)	91.55 ± 1.97	94.44 ± 2.93	96.66 ± 1.67	92.66 ± 1.01	93.11 ± 0.58

*Data represented as means ± SE (*n* = 30). Within rows, means with different superscripts a, b, c, and d indicating that their corresponding means are significantly different at *P* < 0.05.*

### Hematological Parameters

Values of RBCs, WBCs, Hb, and PCV of the experimental fish are shown in Table 4. These parameters significantly (*P* < 0.05) increased in the following ascending order: Sanolife PRO-F^®^; Yeast Plus^®^; Biotronic Top^3^ and finally Digestarom^®^ groups. The lowest RBCs, WBCs, Hb, and PCV levels (*p* ≤ 0.05) were observed in the control diet.

**TABLE 4:** Changes in hematological parameters of Nile tilapia, *O. niloticus* fed on diets with commercial immunostimulants for 8 weeks.

Items	Control	Yeast Plus	Sanolife PRO-F	Biotronic Top^3^	Digestarom^®^
White blood cells (×10^3^ μL^–1^)	42.70^*c*^ ± 0.55	70.90^*b*^ ± 1.93	79.16^*a*^ ± 1.82	66.30^*b*^ ± 1.51	64.13^*b*^ ± 2.06
Red blood cells (×10^6^ μL^–1^)	1.42^*c*^ ± 0.05	2.35^*ab*^ ± 0.06	2.49^*a*^ ± 0.09	2.19^*ab*^ ± 0.02	2.11^*b*^ ± 0.06
Hemoglobin (g dl^–1^)	7.40^*c*^ ± 0.06	13.19^*ab*^ ± 0.17	13.45^*a*^ ± 0.27	12.55^*b*^ ± 0.15	12.30^*b*^ ± 0.27
Packed cell volume (%)	17.89^*c*^ ± 0.12	27.11^*a*^ ± 0.12	28.06^*a*^ ± 0.15	26.84^*a*^ ± 0.03	21.51^*b*^ ± 0.56

*Data represented as means ± SE (*n* = 15). Within rows, means with different superscripts a, b, c, and d indicating that their corresponding means are significantly different at *P* < 0.05.*

### Immune Parameters

**FIGURE 2:**
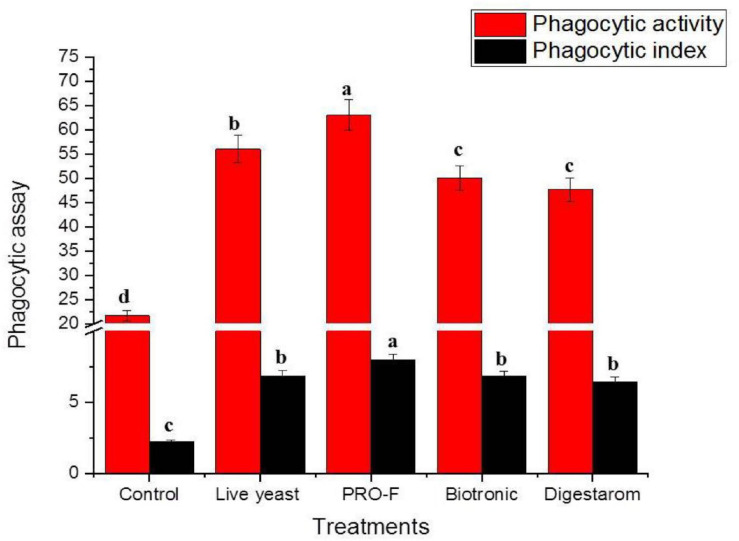
Phagocytic activity % and phagocytic index of Nile tilapia, *O. niloticus*, fed diets with commercial immunostimulants for 8 weeks. Bars superscripted by different letters within the same axis are significantly different (*P* < 0.05), (*n* = 15; means ± SE).

**FIGURE 3:**
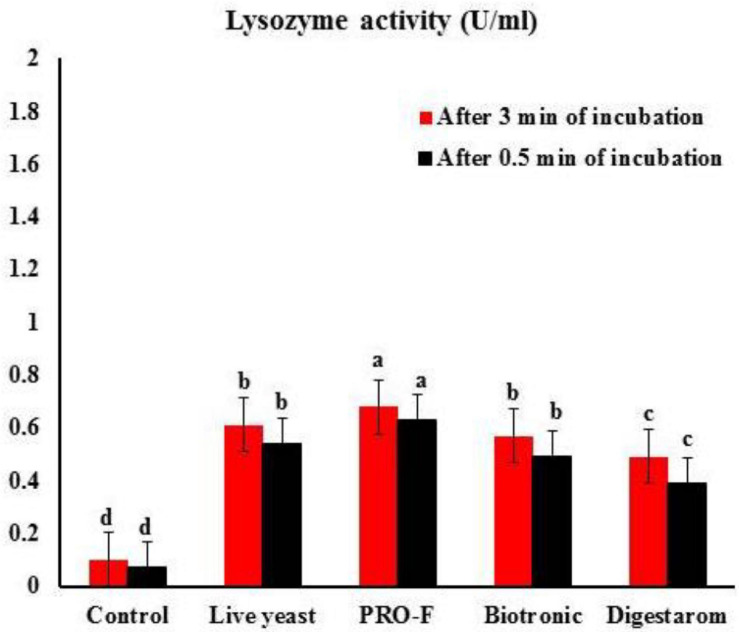
Lysozyme activity after 0.5 min and 3 min of incubation in Nile tilapia, *O. niloticus* fed diets with commercial immunostimulants for 8 weeks. Bars superscripted by different letters within the same axis are significantly different (*P* < 0.05), (*n* = 15; means ± SE).

It is observed that phagocytic activity as well as phagocytic index in experimental fish supplemented with Sanolife PRO-F^®^ were significantly highest as their values were 63.00 ± 1.00% and 7.98 ± 0.03, respectively, compared to all other treatments (Figure 2), Additionally, serum lysozyme in fish supplemented with Sanolife PRO-F was significantly higher compared to all other treatments after 0.5 and 3 min of incubation in *O. niloticus* (Figure 3).

**FIGURE 4:**
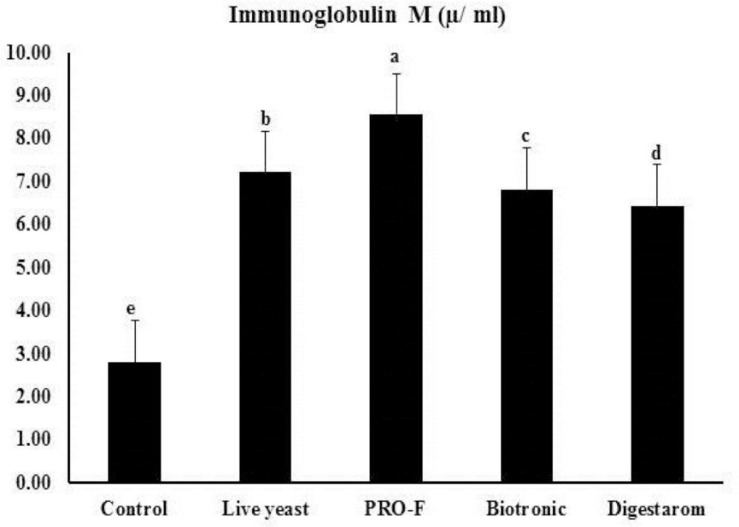
Immunoglobulin M (μ ml^– 1^) activity of Nile tilapia, *O. niloticus* fed diets with commercial immunostimulants for 8 weeks. Bars superscripted by different letters within the same axis are significantly different (*P* < 0.05), (*n* = 15; means ± SE).

It is worth noting that total protein, and globulin showed significant increased values in fish group fed diet supplemented by Sanolife PRO-F^®^ (Table 5). In regard to the IgM levels in Nile tilapia, *O. niloticus*, the best values were obtained in the group supplemented with Sanolife PRO-F^®^, compared to the other fish groups (Figure 4).

**TABLE 5:** Serum total protein, albumin, globulin, and Alb./Glob. ratio in Nile tilapia, *O. niloticus* fed on diets with commercial immunostimulants for 8 weeks.

Items	Control	Yeast Plus^®^	Sanolife PRO-F^®^	Biotronic^®^ Top^3^	Digestarom^®^
Total protein (g dl^–1^)	4.40^*e*^ ± 0.02	5.87^*b*^ ± 0.02	6.91^*a*^ ± 0.08	5.43^*c*^ ± 0.03	5.19^*d*^ ± 0.02
Albumin (g dl^–1^)	2.68^*a*^ ± 0.01	2.14^*d*^ ± 0.01	1.70^*e*^ ± 0.05	2.21^*c*^ ± 0.03	2.33^*b*^ ± 0.02
Globulin (g dl^–1^)	1.72^*e*^ ± 0.01	3.73^*b*^ ± 0.01	5.21^*a*^ ± 0.16	3.22^*c*^ ± 0.03	2.86^*d*^ ± 0.01
Alb./glob. ratio	1.56^*a*^ ± 0.02	0.57^*b*^ ± 0.01	0.33^*c*^ ± 0.03	0.69^*b*^ ± 0.02	1.64^*a*^ ± 0.02

*Data represented as means ± SE (*n* = 15). Within rows, means with different superscripts a, b, c, and d indicating that their corresponding means are significantly different at *P* < 0.05.*

### Antioxidants Enzyme Assay

**FIGURE 5:**
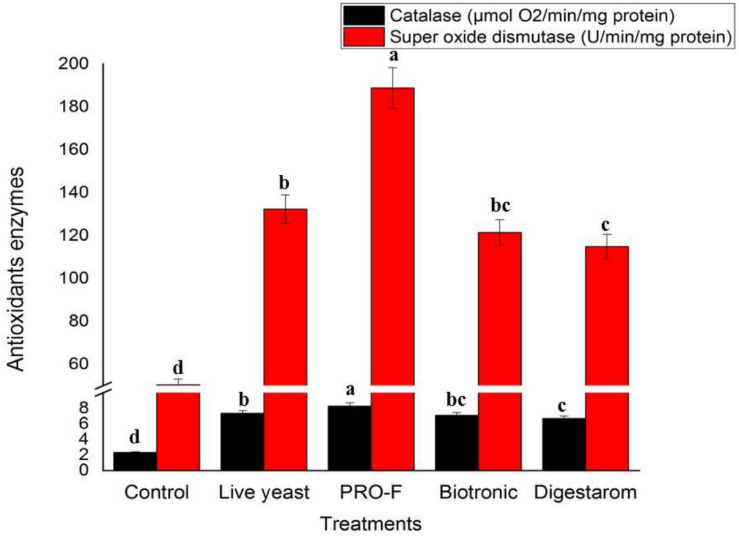
Activities of serum CAT and SOD of Nile tilapia, *O. niloticus* fed diets with commercial immunostimulants for 8 weeks. Bars superscripted by different letters within the same axis are significantly different (*P* < 0.05), (*n* = 15; means ± SE).

The best results for CAT and SOD levels were recorded in Nile tilapia, *O. niloticus* supplemented with Sanolife PRO-F (Figure 5). The lowest values were observed in the control fish group.

### Morphometric Analysis of Intestinal Tract

**FIGURE 6:**
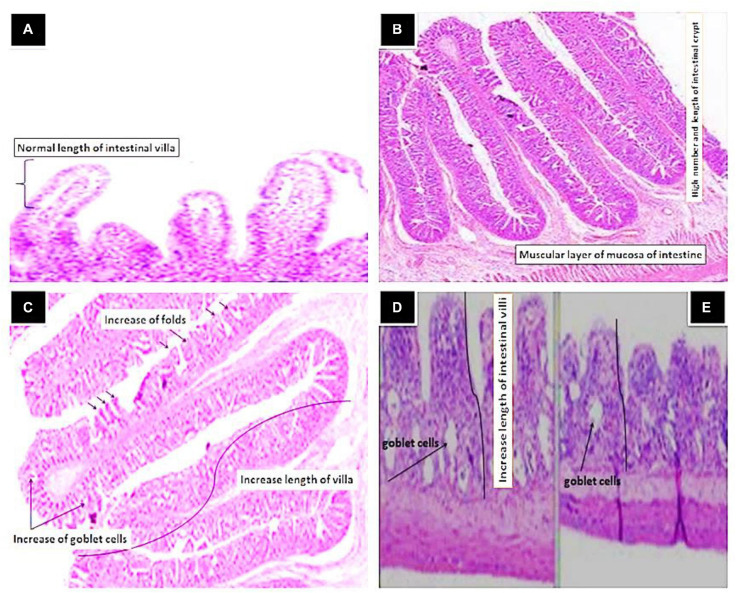
Histological section of Intestinal tract of Nile tilapia, *O. niloticus* fed Control **(A)** showing normal length of intestinal villi and crypt (H&E × 20), Yeast Plus^®^ **(B)** showing in length of villi and mucosal folds and high number of intestinal crypt (Arrow), Sanolife PRO-F^®^ **(C)** showing increase of the mucosal folds and high numbers of goblet cells as well as increase in length of intestinal villi and high number of intestinal crypt (Arrow), Biotronic^®^ Top^3^ **(D)** and Digestarom^®^ **(E)** showing slight activation of goblet cells in the lamina propria and slight increase length of intestine villi (Arrows) (H&E × 20).

The results of the morphological analysis of intestinal villi height/width, crypt depth as well as number of goblet cells are summarized in Table 6 and Figures 6A–E. The best intestinal villi height/width, crypt depth, and number of goblet cells were recorded in fish groups supplemented with Sanolife PRO-F^®^, Yeast Plus^®^, and Biotronic^®^ Top^3^. The epithelium lining the intestine was simple columnar cells, which contain enterocytes, goblet cells and scattered ciliated cells. The length of the intestinal villi, in the anterior and terminal parts of the intestine, was significantly increased with the Sanolife PRO-F^®^ and live Yeast^®^ groups. The number of PAS-positive goblet cells was also significantly increased in the Sanolife PRO-F^®^ and Yeast Plus^®^ groups.

**TABLE 6:** Morphometric analysis of intestinal tract in Nile tilapia, *O. niloticus* fed diets with commercial immunostimulants for 8 weeks.

Items	Control	Yeast Plus^®^	Sanolife PRO-F^®^	Biotronic^®^ Top^3^	*Digestarom*^®^
Villi height (μm)	266.67^*c*^ ± 11.60	401.00^*ab*^ ± 7.81	417.33^*a*^ ± 3.84	383.67^*ab*^ ± 7.53	374.33^*b*^ ± 6.93
Villi width (μm)	32.33^*d*^ ± 3.52	59.33^*ab*^ ± 2.33	62.00^*a*^ ± 2.51	46.00^*c*^ ± 3.21	47.00^*bc*^ ± 1.73
Area of absorption (μm^2^)	8585^*c*^ ± 802.3	23828^*a*^ ± 1389.7	25887^*a*^ ± 1227.3	17667^*b*^ ± 1424	17583^*b*^ ± 583.7
Crypt depth	38.66^*c*^ ± 3.17	70.66^*ab*^ ± 3.48	77.66^*a*^ ± 2.96	64.33^*ab*^ ± 2.90	58.00^*b*^ ± 2.64
Goblet cells	*++*	*+++*	*+++*	*++*	*++*

*++: Normal number of goblet cells, +++: Moderate number of goblet cells. Data represented as means ± SE (*n* = 15). Within rows, means with different superscripts a, b, c, and d indicating that their corresponding means are significantly different at *P* < 0.05.*

## Discussion

The current study evaluated the use of different immunostimulants as dietary supplementations for *O. niloticus* on growth performance, total production Kg m^–3^, and immune response.

### Water Quality Assessments

In the current study, feed additives of PRO-F^®^ and Yeast Plus^®^ treatments showed significant improvements in all water quality parameters as compared with the control ponds. These findings may be due to certain probiotic mixtures of *Bacillus* sp., which generate groups of exogenous enzymes that broken away digested nutrients (Farzanfar, 2006; Liu L. et al., 2012; Tran et al., 2016). Furthermore, the use of healthy microbes (bacteria) and plankton in fish culture helps to improve water quality parameters, that provides a good environment for increasing rates of growth and maintaining fish health (El-Haroun et al., 2006; Gobi et al., 2018; Joffre and Verdegem, 2019; Abdel-Tawwab et al., 2020b). In addition, the microbiota excreted with fish feces contribute to improved positive bacterial species that strengthen the parameters of water quality (Verschuere et al., 2000; Balcázar et al., 2006; Bosma and Verdegem, 2011).

### Growth Performance and Feed Utilization

According to the current study, the highest increase in growth performance and total production per m^3^ were recorded in fish feed on diets supplemented with PRO-F followed by live yeast. These findings show that both immunostimulants have a beneficial impact on growth efficiency rates and make aquaculture more sustainable (Froese, 2006; Sun et al., 2010; Yossa et al., 2018). The obtained results may be due to the increase of digestive enzymes that contribute to improved nutrition and enhanced intestinal absorption, thereby boosting fish growth (Abdel-Tawwab et al., 2008, 2020b; Abdel-Tawwab and Ahmad, 2009; Abdel-Tawwab, 2012).

### Hematological Parameters

Blood measurements are indeed a crucial factor in determining nutritional suitability, concentrations and toxicity, as well as their impact on the circulatory system in aquatic animals (Hari et al., 2004; Dash et al., 2015). In the current study, all blood parameters; RBC, WBC, Hb, and PCV, were improved by feed additives of probiotics, prebiotics, and organic acids compared to the control diet without supplements. The hematological parameters were improved during experiment as they were affected by feed additives (Nayak et al., 2007; Ramesh et al., 2015). It is clearly evident that there is an increase in hemoglobin, and hematocrit in Nile tilapia, *O. niloticus*, which fed on *Bacillus amyloliquefaciens* (Reda and Selim, 2015), as well as increased white blood cell in *Labeo rohita* (Ham.) which subjected on *Bacillus subtilis* diets (Kumar et al., 2008).

### Immune Parameters

Immunostimulants supplementation has been shown to improve fish immunity by stimulating cellular and muscular immune function (Kim et al., 2015; Kuebutornye et al., 2019). These results were confirmed through the immunological aspect of blood lysosome activity, total protein, globulin, immunoglobulin M, and antioxidative enzymes of *O. niloticus*. In general, phagocytic activity and index were improved when fish were fed on probiotics, prebiotics, and organic acids containing diets compared to the control fish, due to the antimicrobial compounds generated from the immunostimulants (Rahman et al., 2018), and to the improve of the natural complement, phagocytic activities, and serum peroxidase (Salinas et al., 2008; Ramesh et al., 2015). These results potentially indicate to the contribution of *Bacillus* and yeast-based probiotic administration in enhancing and promoting the phagocytic activity as well as the phagocytic index of Nile tilapia (Telli et al., 2014; Liu et al., 2017).

Lysozyme is a basic humoral immune defense factor typically developed in aquatic animals when it targets bacterial peptidoglycans, often Gram-positive bacteria, in the cell membrane, culminating in the stimulation of bacterial phagocytosis by phagocytic cells (Magnadottir et al., 2005; Selim and Reda, 2015). In addition, Zhou et al. (2010) found that the probiotics improved lysozyme activity in the probiotic group with a significant difference higher than in the control group. The present findings revealed a substantial improvement significantly in lysozyme. These findings align with Aly et al. (2008) and Liu C.H. et al. (2012) who discovered that fish fed on diets supplemented by *Bacillus* sp. was substantially higher lysozyme activity than those in fish fed a control diet for 4 weeks. Studies using yeast as a dietary additive to strengthen the innate immunity of Nile tilapia also support these findings (Abdel-Tawwab et al., 2008, 2020b; Abdel-Tawwab, 2012; Khalila et al., 2018).

Serum parameters are considered to become the main factor in evaluating the immune function and immune system response in fish species (Mulcahy, 1971; Van Hai, 2015). It is worth noted that total proteins, globulin, and immunoglobulin M levels showed increased values in the groups fed on diets with probiotics and prebiotics at the end of the experiment. Likewise, there is an improvement of globulin seen in probiotics dependent on Bacilli (Reda and Selim, 2015) and yeast–based diets that fed to Nile tilapia (Abdel-Tawwab et al., 2008, 2020a, 2020b; Abdel-Tawwab, 2012). Microorganisms used throughout the tilapia culture have been shown to be successful improving in the autoimmune reaction, including such *B. subtilis*, *Saccharomyces cerevisiae*, *Lactobacillus acidophilus*, and *Bacillus amyloliquefaciens* (Abdel-Tawwab et al., 2008; Selim and Reda, 2015; Abou-El-Atta et al., 2019; Kuebutornye et al., 2019).

### Antioxidants Activity

The results revealed that CAT and SOD activity significantly increased in fish groups fed on diets supplemented with probiotics and prebiotics. Similarly, Guardiola et al. (2016) found that the antioxidant activity of *Pediococcus acidilactici* was observed to cause regulation of the CAT and SOD activities as feed supplements in aqua-feeds. These results may be due to functions by releasing stopped oxygen levels (ROS) free radicals, inducing oxidative stress which can interrupt and influence metabolic processes and thereby destroy cell groups. Each condition that raises the accumulation of ROS is considered oxidative stress, which can contribute to the disturbance and control of mitochondrial function and thereby damage cell components (Abdel-Tawwab and Wafeek, 2017; Mansour et al., 2017; Gobi et al., 2018).

### Morphometric Analysis of Intestinal Tract

Gut morphology is a crucial factor affected by immunostimulant diets and regulates metabolic physiology, resulting in improved digestion and absorption of nutrients, resulting in improved growth efficiency in fish (Cerezuela et al., 2013; Hoseinifar et al., 2014; Adeshina et al., 2019; Sewaka et al., 2019). In the current research, the progress of intestinal morphometrics led to the increased digestion and absorption of nutrients after feeding on probiotics, prebiotics, and organic acids as dietary feed supplements. These indicate to a rise in the region of enterocyte absorption contributing to improved growth efficiency (Ferguson et al., 2010; Giatsis et al., 2016). The present study indicated that the best morphometry of the intestine, crypt depth, and goblet cell count, in current the experiment, were recorded in the fish groups fed on diets supplemented with PRO-F^®^ and Yeast Plus^®^. The elevation of intestinal villus height/length relative to the control group showed a statistically significant improvement that could increase surface intestinal absorption, boost nutrient absorption, and consequently improve growth efficiency (Sun et al., 2010; Pirarat et al., 2011; Yossa et al., 2018; Khalil et al., 2019).

## Conclusion

The current study found an improving effects on growth performance, nutrient utilization, hemato-biochemical, non-specific immune parameters, antioxidant enzyme, and the intestinal morphometric of Nile tilapia, *O. niloticus* fed on a diet supplemented with Sanolife PRO-F^®^ or Yeast Plus^®^ at 0.5 or 1.0 kg ton^–1^, respectively.

## Data Availability Statement

The raw data supporting the conclusions of this article will be made available by the authors, without undue reservation.

## Ethics Statement

The animal study was reviewed and approved by the (ALEXU-IACUC) Institutional Animal Care and Use Committee AU: 14/07/20/2/7. Written informed consent was obtained from the owners for the participation of their animals in this study.

## Author Contributions

MK: data curation, methodology, software, visualization, writing, and original draft preparation. TS: investigation, monitoring, and supervision. EO: investigation, monitoring, and reviewing. AF: conceptualization and reviewing. AN: conceptualization, supervision, software, and validation. HK: conceptualization, data creation, writing- original draft preparation, and reviewing and editing. All authors contributed to the article and approved the submitted version.

## Conflict of Interest

The authors declare that the research was conducted in the absence of any commercial or financial relationships that could be construed as a potential conflict of interest.
